# Does It Really Take 17 Years for Clinical Trial Results to Reach Clinical Practice? A Meta‐Research Protocol of the Evaluation of Trials Informing Recommendations

**DOI:** 10.1002/gin2.70069

**Published:** 2026-04-10

**Authors:** Brian S. Alper, Joanne Dehnbostel, Rachel Couban, Kenneth J. Wilkins, Karen A. Robinson, Harold Lehmann, Scientific Knowledge Accelerator Foundation

**Affiliations:** ^1^ Computable Publishing LLC Franklin North Carolina USA; ^2^ Scientific Knowledge Accelerator Foundation Franklin North Carolina USA; ^3^ Mel and Enid Zuckerman College of Public Health University of Arizona Tucson Arizona USA; ^4^ Department of Anesthesia McMaster University Hamilton Ontario Canada; ^5^ Biostatistics Program/Office of Clinical Research Support, Office of the Director, National Institute of Diabetes & Digestive & Kidney Diseases, National Institutes of Health (NIH/NIDDK/OD) Bethesda Maryland USA; ^6^ Department of Preventive Medicine & Biostatistics F. Edward Hébert School of Medicine at the Uniformed Services University of the Health Sciences Bethesda Maryland USA; ^7^ Department of Medicine School of Medicine, Johns Hopkins University Baltimore Maryland USA; ^8^ Department of Health Policy and Management and Department of Epidemiology Johns Hopkins Bloomberg School of Public Health, Johns Hopkins University Baltimore Maryland USA

**Keywords:** bibliometrics, Bibliometrics MeSH code (D015706), clinical guideline, Clinical Guideline as topic code (D017410), evidence based medicine, Evidence Based Medicine (D019317), meta‐research, Meta‐Research MeSH code (D000098346), randomised controlled trials, Randomised Controlled Trials as topic (D016032)

## Abstract

**Introduction:**

It has been estimated to take 17 years for research to change clinical practice. Scientific Knowledge Accelerator Foundation aims to narrow this research‐practice gap, hypothesising that making scientific evidence computable will accelerate the pace of knowledge transfer. We designed this study to develop a reusable process for measuring the rate of knowledge transfer from clinical trials to practice guidelines to evaluate the effectiveness of our efforts in the years to come.

**Methods:**

Our study uses citation in clinical guidelines as a proxy for use of research in practice. We will evaluate a convenience sample of randomised controlled trials (RCTs), trace their citations in the clinical literature, and measure the length of time it takes for RCT results to be incorporated into recommendations for clinical practice. For each ‘index article’ in our convenience sample, we will record the date its results were publicly available (including preprint date and date of posting results in a registry, if applicable). Then, we will identify articles that cite the index article, sorting by date to find the earliest use of the RCT findings to inform an evidence‐based recommendation for clinical practice. To qualify, these articles will meet three criteria: (1) systematically derived, (2) incorporating the RCT in the results section of the article and (3) making a recommendation intended to inform clinical practice. Review articles meeting the first two criteria will be checked for their use in subsequent generation of recommendations.

**Discussion:**

This study will develop a process to approach the question, ‘How long does it take for results of clinical trials to be incorporated into published systematically derived recommendations intended to guide clinical practice?’

## Summary

- It is frequently reported to take 17 years for clinical research to change practice.
- The time from research to practice has not been reproducibly measured in the current century.
- This study will measure the time from the availability of research results to their inclusion in clinical practice recommendations.
- This study protocol offers a reusable process for collaborative and independent testing.

## 1 Introduction

It has been estimated to take 17 years for results from clinical research to be incorporated into clinical practice. However, estimates of the pace of scientific knowledge transfer vary. Each using a different method, these estimates span multiple time intervals, including 6–13 years from publication of clinical trials [1, 2] to use in guideline recommendations [3], 0–49 years (median 8 years) from publication to guidelines [4], 9–14 years (mean 13 years) from publication to guidelines [5], and 11–67 years (mean 28 years) from enabling scientific research to drugs reaching the market [6].

We do not know if the 17‐year estimate, or any of the other estimates, is still accurate with the evolution of the internet. We also do not know if the rate of knowledge transfer from research to practice is influenced by major events such as the introduction of Google in 2000, the publication of the Institute of Medicine's ‘Clinical Practice Guidelines We Can Trust’ in 2011 [7] and the COVID‐19 pandemic in 2020.

We also do not know if efforts to develop standards for data exchange of evidence will make a difference in the rate of knowledge transfer from research to practice. In 2011, Health Level 7 International (HL7) introduced *Fast Healthcare Interoperability Resources (FHIR)®* as a standard for data exchange (a set of data models, data formats, data elements, and API protocols). FHIR is being adopted widely for health data exchange [8, 9, 10]. In 2018, participants from the communities of biomedical/healthcare research, systematic review development, clinical practice guideline development and clinical decision support development collaborated to obtain approval of HL7 to extend FHIR to evidence‐based medicine (EBM) knowledge assets, a project now called EBMonFHIR [11]. The community supporting the EBMonFHIR project evolved to become the *Health Evidence Knowledge Accelerator (HEvKA)* in 2023, an open collaborative effort with 11 active working groups meeting weekly and bringing together at least 100 people from many disciplines and 28 countries [12, 13]. In 2021, a dozen of the most active HEvKA participants from six countries established a nonprofit organisation called the Scientific Knowledge Accelerator Foundation (SKAF) [14].

To determine whether our efforts are effective at progressing toward a state of health care where research moves more quickly into practice, SKAF designed this study to develop a reproducible method to measure the rate of scientific knowledge transfer from clinical trials to practice guidelines.

## 2 Methodology

We will conduct a meta‐research study with a citation analysis of clinical trials and have outlined the major concepts in our study in accordance with the STROBE guidelines [15].

### 2.1 Study Design

For a cohort of published articles reporting research results (‘index articles’), we will measure the length of time it takes for the findings to be incorporated into recommendations for clinical practice, using time‐to‐event estimation methods.

### 2.2 Setting

To target our efforts efficiently, we selected a convenience sample of articles known to be incorporated into a clinical guideline, specifically a cohort of 159 index articles reporting randomised controlled trials (RCTs) of erythropoiesis‐stimulating agents (ESAs) in patients with chronic kidney disease (CKD). This group of articles was originally selected for inclusion in systematic reviews for the Kidney Disease Improving Global Outcomes (KDIGO) Guidelines for Anaemia in CKD [16] (see Appendix S2).

### 2.3 Index Article Variables

**Figure 1:**
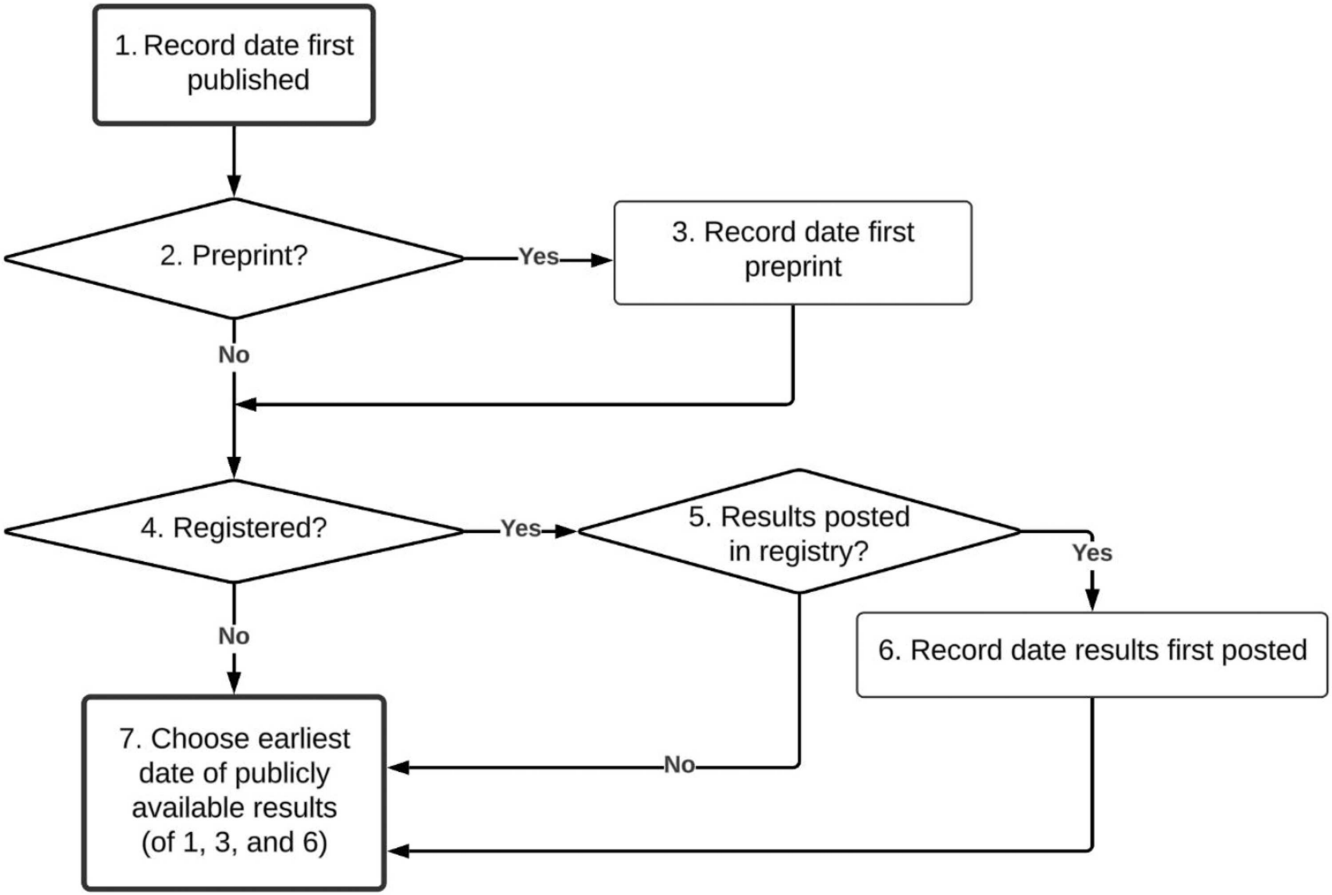
Flowchart for establishing the earliest date of publicly available results. Bolded borders indicate start and end.

For each index article, we will extract or determine the following variables:

1. Date the article was first published (publicly available). This article will be represented with a unique identifier, the FEvIR Object Identifier (FOI) for a Citation Resource.
2. Whether a preprint was published.
3. If a preprint was published, earliest date when a preprint was published and that preprint gets an FOI for a Citation Resource.
4. Whether the study was registered in a study registry (If the study was registered in a study registry, record the name of the study registry and the identifier for the study.).
5. If the study was registered in a study registry, whether results were posted to the study registry.
6. If the study results were posted to a registry, earliest date when results were publicly available and the results posting gets an FOI (either for a Citation Resource or a Composition Resource as may be used for ClinicalTrials.gov results).
7. Primary Datapoint A (derived from the above), date the study results were first publicly available (earliest of 1, 3 or 6) (see Figure 1).
8. Whether this article is a report of a trial that is reported in one of the other 158 sample articles (used to combine multiple reports from the same study, to avoid spurious associations from network dependence).
9. If this article is a report of a trial included in the other 158 sample articles, the identifier(s) of the other articles.
10. Journal name (additional bibliometric data points that can be automatically extracted from the citation records, for exploratory analyses).
11. Language (additional bibliometric data points that can be automatically extracted from the citation records, for exploratory analyses).

For variables 1–6, an investigator will view the full text of the index article to identify dates and mentions of preprints or study registration. If preprints or study registration is noted, then the investigator will check those sources as feasible. For each of these observations, two investigators will independently record their findings, and any data mismatches will be discussed for final determination. Variable 7 will be calculated.

For variables 8–9, the citation records for the 159 index articles will be checked for titles with matching acronyms and author lists with matching names. In addition, any articles with the same study registry identifier will be recognised from the data collected for variable 5.

Variables 10–11 will be extracted from the bibliographic records.

Retrospective guideline recommendation mapping

Guidelines relevant to the use of ESAs in CKD will be identified through searches of guideline registries, DynaMed, PubMed and Google. Guideline registries will include those of the Guidelines International Network (GIN), Arbeitsgemeinschaft der Wissenschaftlichen Medizinischen Fachgesellschaften (AWMF, the German association of medical scientific societies), and the Medical Information Network Distribution Service (MINDS).

For each guideline, we will extract or determine the following data:

1. Date the guideline was first published (publicly available). This guideline will be represented with a unique identifier, the FOI for a Citation Resource or Composition Resource (if the guideline is available in FHIR form).
2. The list of all results from RCTs which contributed to the recommendations, in the form of FOIs. Each guideline will be searched for reference lists for direct references, as well as references to systematic reviews or meta‐analyses which will in turn be search for contributing RCTs. Data sources other than RCTs will not be mapped.

Prospective guideline recommendation mapping

For each index article, we will record any guideline FOIs for which the results FOIs are found in the retrospective guideline recommendation mapping.

To identify the earliest date that a recommendation for clinical practice was published (publicly available) in which the RCT results were incorporated into the information used to create or justify the recommendation, we will:

1. All articles citing the index article will be identified using Scopus [17].
2. Starting with the oldest, articles citing the index article will be evaluated to determine:
  a. Whether the article meets our eligibility criteria (systematically derived, incorporating the index article in its results).
    i. ‘Systematically derived’ means that, for the methods used to select evidence for consideration, there is a specification of search sources and inclusion criteria.
    ii. ‘Incorporating the index article in its results’ means that the clinical trial results are incorporated into the Results of the citing article and not just the Introduction and Discussion (if the citing article has focused Results).
  b. If the article meets our eligibility criteria, the date the article was first published.
  c. If the article meets our eligibility criteria, whether the article includes a recommendation intended to inform clinical practice.
  d. If the article meets our eligibility criteria but does not include a recommendation intended to inform clinical practice, then the article becomes a ‘secondary index article’ and steps 1–2 will be repeated using the ‘secondary index article’ as the index article.
3. The tracing of citations of index articles will continue until the earliest inclusion in a recommendation intended to inform clinical practice is found. Once a date for inclusion in a recommendation is identified, then citations after that date do not need to be checked.

For each index article, we will extract or determine the following variables:

12. Primary Datapoint B, earliest date that a recommendation for clinical practice was published (publicly available) in which the study results were incorporated into the information used to create or justify the recommendation (described above).
13. Primary Outcome *Time to integration of results in a recommendation,* calculated by measuring the duration of time elapsed between Primary Datapoint A and Primary Datapoint B.
14. Primary Outcome Event‐type Indicator, to characterise how to analyse the Primary Outcome, which indicates whether the event (Primary Datapoint B) is actually the timing of publication of a recommendation or the timing of last observation without integration of results in a recommendation. This ‘censoring’ or ‘competing event’ indicator is not required in our protocol (as all index articles are known to be integrated in a recommendation) but is included here for completeness for a reusable methodology.

Variable 13 (Primary Outcome), the *Time to integration of results in a recommendation*, will be calculated as the duration of time between the Primary Datapoints (A: Date the RCT results were first publicly available, B: Earliest date that a recommendation for clinical practice was published (publicly available) in which the RCT results were incorporated into the information used to create or justify the recommendation) for each of the articles in the sample for analysis that have both Primary Datapoints. The *Time to integration of results in a recommendation* will be calculated with a unit of months, assuming the 15th of the month when days are not stated and the sixth month of the year when months are not stated.

### 2.4 Statistical Methods

For the primary outcome, the *Time to integration of results in a recommendation*, we will report a time‐to‐event analysis to include nonparametric distributional summaries such as Kaplan–Meier (alternatively, cumulative incidence function) estimates and multivariable models that characterise the time‐to‐event distribution features' dependence on covariates, such as (cause‐specific) hazards‐based modelling via Cox regression and its extensions. We will perform a cluster analysis in Cox regression using a robust sandwich variance estimator to account for correlated data (index articles derived from the same study) as the primary analysis.

Sensitivity analyses will explore a range of approaches to accommodate serial and other forms of dependence among the observations, including use of co‐citation networks or bibliographic couplings, and alternate secondary‐estimand regression modelling (e.g., accelerated failure time and additive hazards). Descriptive statistics will include the mean, standard deviation, median, range, and interquartile range, stratified by covariates.

Supplementary analyses will include reporting without the use of preprints and trial registries.

Exploratory time‐to‐event analyses will consider the influence of bibliographic factors (year of publication, journal name, language) on the *Time to integration of results in a recommendation*. The year of publication will be analysed in four categories (before 2000, 2000–2011, 2012–2019, after 2019). As supplementary analyses to this analytic choice, we may explore more flexible data‐driven approaches to incorporate year of publication, such as penalised splines with corresponding degree of smoothing determined by the mixed‐effects model method of fitting.

A Statistical Analysis Plan will be developed to accompany this protocol and posted with a computable expression of this protocol at https://fevir.net/resources/ResearchStudy/282930#json.

### 2.5 Research Software

The research will be supported with the *Fast Evidence Interoperability Resources (FEvIR®) Platform*, created by Computable Publishing LLC [18, 19], which provides an online platform for data entry, conversion, storage, and retrieval for scientific knowledge in structured form matching the Evidence Based Medicine on FHIR Implementation Guide (EBMonFHIR IG). The EBMonFHIR IG is a technical specification providing a standard for interoperable data exchange for clinical research methods, results (evidence), and evidence‐based guidance.

Tools on the FEvIR Platform that will be used for this research include:

- FEvIR®: RAte of Dissemination Assessment Research (RADAR) Tool facilitates research conduct and project management for an investigation to measure the rate of scientific knowledge transfer.
- FEvIR®: Citation Builder creates a Citation Resource. FEvIR®: Citation Viewer displays a Citation Resource.
- Computable Publishing®: MEDLINE‐to‐FEvIR Converter converts PubMed MEDLINE XML to FHIR® JSON.
- Computable Publishing®: RIS‐to‐FEvIR Converter converts data with an RIS format into FEvIR Resources in FHIR Citation JSON.
- Computable Publishing®: ClinicalTrials.gov‐to‐FEvIR Converter converts ClinicalTrials.gov records to FHIR® JSON.

We will use statistical software such as SAS (PROC LIFETEST, LIFEREG, COXPH) or R (survival, survminer, cmprsk packages) with auxillary use of non‐statistical software for network‐ and bibliometric‐geared analyses such as neo4J (https://github.com/neo4j‐graph‐examples/citations), Sci2 (https://sci2.cns.iu.edu/user/index.php), or iCite (https://icite.od.nih.gov/analysis).

We will use Scopus to find bibliographic citations.

## 3 Discussion

### 3.1 Bias and Limitations

Use of a convenience sample as the cohort of index articles could introduce an availability bias and potentially miss evaluation of relevant trials. The systematic search and selection process in the KDIGO guideline is assumed to be comprehensive for the trials relevant to the topic of ESAs for CKD.

Use of index articles known to be incorporated into a guideline does not reflect the typical scenario in which any given research result may or may not be incorporated into a guideline at the time of analysis. In future use of this methodology, careful application of time‐to‐event methods (with accommodation of censoring and competing events) is warranted [20].

Observations from non‐independent sample subjects (i.e., each results publication) can result in spurious associations from the latent presence of network dependence [21]. To mitigate this, multiple reports from the same clinical trial will be combined to be treated as a single article for analysis; results' residual dependence from distinct trials will be explored within sensitivity analyses via plausible extensions to time‐to‐event regression that account for serial/co‐citation networks. The deduplicated combined set of index articles will become the sample for primary analysis.

The *Time to integration of results in a recommendation* may be dependent on any number of measurable and unmeasurable factors. Three potential factors (year of publication, journal name, language) are measurable in our research dataset.

The use of a bibliometric database (Scopus) to identify uses of a clinical trial report may miss uses that do not follow traditional publication practices, such as publication through the grey literature or unconventional representation of citations. If the current practice of publishing RCTs in journal articles is supplanted by new methods of scholarly communication, our method might need to adapt or it will become obsolete.

Finally, we are using a proxy measure for the use of RCT results in clinical practice. Using incorporation into a guideline as our primary outcome measure may not accurately reflect incorporation of RCT results into clinical practice. Guidelines may not be implemented or adhered to in clinical practice, and pathways other than guidelines, such as marketing or medical education, may result in application of RCT results without guidelines.

As a single study, the results are not generalisable to other clinical areas and are not generalisable to other interventions for chronic kidney disease. However, once demonstrated, the study method could be repeatedly applied to address other clinical domains.

## 4 Conclusion

The results of this study will introduce a new and feasible measure for the rate of scientific knowledge transfer from clinical trials to practice guidelines. This study will also apply and demonstrate a method for measuring the rate of scientific knowledge transfer from clinical trials to clinical practice guidelines. This new method can be tested by SKAF and others for reproducibility, and derivatives of this method can be developed for other types of scientific knowledge transfer. A reproducible method could be scaled with one or more large language models, such as ChatGPT, in workflow contexts such as Retrieval Augmented Generation. SKAF welcomes participation through open weekly meetings, including the Measuring the Rate of Scientific Knowledge Transfer Working Group. Participation details can be found at fevir.net/HEvKA.

## Author Contributions

**Brian S. Alper:** conceptualization, methodology, project administration, writing – original draft, writing – review and editing. **Joanne Dehnbostel:** conceptualization, methodology, project administration, writing – review and editing. **Rachel Couban:** conceptualization, methodology, writing – review and editing. **Kenneth J. Wilkins:** conceptualization, methodology, writing – review and editing. **Karen A. Robinson:** conceptualization, methodology, writing – review and editing. **Harold Lehmann:** conceptualization, methodology, visualization, writing – review and editing.

## Funding

The authors received no specific funding for this work.

## Conflicts of Interest

Alper owns Computable Publishing LLC. Dehnbostel is an employee of Computable Publishing LLC. Alper, Dehnbostel, Couban, and Robinson are board members for the Scientific Knowledge Accelerator Foundation. Wilkins is an employee of the US Federal government, Department of Health and Human Services, with an adjunct academic appoint to the F. Edward Hébert School of Medicine at the Uniformed Services University of the Health Sciences, Military Health Agency.

## Supporting information


**Appendix 1:** Scientific Knowledge Accelerator Foundation Board Members. **Appendix 2:** Articles included in sample.

## Data Availability

There are no data to share for the study protocol. Study details are available on the FEvIR Platform at https://fevir.net/resources/ResearchStudy/282930. The study data will be openly shared at https://fevir.net.
