# Supplementary material for: Does It Really Take 17 Years for Clinical Trial Results to Reach Clinical Practice? A Meta‐Research Protocol of the Evaluation of Trials Informing Recommendations
Source: Clin Public Health Guidel. 2026 Apr 10;3(2):e70069. doi: 10.1002/gin2.70069 (PMC13563394; doi:10.1002/gin2.70069)
Supplement: Supplementary file 1 [file GIN2-3-e70069-s001.docx]

Supplementary Materials for

**Title:**

**Does it really take 17 years for clinical trial results to reach clinical practice? A meta-research protocol**

**Running Title:**

**Time from research to practice: a protocol**

**Authors:**

Brian S. Alper

Joanne Dehnbostel

Rachel Couban

Kenneth J. Wilkins

Karen A. Robinson

Harold Lehmann

Scientific Knowledge Accelerator Foundation

Appendix 1. Scientific Knowledge Accelerator Foundation Board Members

| Name | Position | Affiliation | Country |
| --- | --- | --- | --- |
| Muhammad Afzal | Board Member | Birmingham City University, Department of Computer Science, Birmingham, UK | UK |
| Brian S. Alper | President | Computable Publishing LLC | USA |
| Noella Awah | Board Member | eBASE Africa | Cameroon |
| Rachel Couban | Board Member | McMaster University | Canada |
| Joanne Dehnbostel | Secretary | Computable Publishing LLC, University of Arizona, Tucson, USA | USA |
| Christopher Enright | Treasurer | Computable Publishing LLC | USA |
| Bhagvan Kommadi | Board Member | Quantica Computacao Private Limited | India |
| Ilkka Kummano | Board Member | Duodecim/EBMEDS | Finland |
| Harold Lehmann | Board Member | Johns Hopkins University | USA |
| Mattias Löbe | Board Member | Leipzig University | Germany |
| Karen Robinson | Board Member | Johns Hopkins University | USA |
| Khalid Shahin | Board Member | Computable Publishing LLC | USA |
| Mario Tristan | Board Member | IHCAI INSTITUTE | Costa Rica |

Appendix 2. Articles included in sample

1.	Agarwal R, Anand S, Eckardt KU, Luo W, Parfrey PS, Sarnak MJ, et al. Overall Adverse Event Profile of Vadadustat for the Treatment of Anemia Associated With Chronic Kidney Disease in Phase 3 Trials. Am J Nephrol. 2022.

2.	Agrawal D, Varade D, Shah H, Nazar A, Krishnan J, Shukla V, et al. Desidustat in Anemia due to Non-Dialysis-Dependent Chronic Kidney Disease: A Phase 3 Study (DREAM-ND). Am J Nephrol. 2022;53(5):352-60.

3.	Akaishi M, Hiroe M, Hada Y, Suzuki M, Tsubakihara Y, Akizawa T. Effect of anemia correction on left ventricular hypertrophy in patients with modestly high hemoglobin level and chronic kidney disease. J Cardiol. 2013;62(4):249-56.

4.	Akiba T, Akizawa T, Kakuma T. Randomized double-blind comparative study of recombinant human erythropoietin (epoetin kappa, produced by serum-free culture) in renal anemia patients on hemodialysis. Japanese Pharmacology and Therapeutics. 2010;38(2):181-98.

5.	Akizawa T, Gejyo F, Nishi S, Iino Y, Watanabe Y, Suzuki M, et al. Positive outcomes of high hemoglobin target in patients with chronic kidney disease not on dialysis: a randomized controlled study. Ther Apher Dial. 2011;15(5):431-40.

6.	Akizawa T, Iwasaki M, Otsuka T, Yamaguchi Y, Reusch M. Phase 3 Study of Roxadustat to Treat Anemia in Non–Dialysis-Dependant CKD. Kidney Int Rep. 2021;6(7):1810-28.

7.	Akizawa T, Iwasaki M, Yamaguchi Y, Majikawa Y, Reusch M. Phase 3, Randomized, Double-Blind, Active-Comparator (Darbepoetin Alfa) Study of Oral Roxadustat in CKD Patients with Anemia on Hemodialysis in Japan. J Am Soc Nephrol. 2020;31(7):1628-39.

8.	Akizawa T, Macdougall IC, Berns JS, Bernhardt T, Staedtler G, Taguchi M, et al. Long-Term Efficacy and Safety of Molidustat for Anemia in Chronic Kidney Disease: DIALOGUE Extension Studies. Am J Nephrol. 2019;49(4):271‐80.

9.	Akizawa T, Nangaku M, Yamaguchi T, Koretomo R, Maeda K, Miyazawa Y, et al. A Phase 3 Study of Enarodustat (JTZ-951) in Japanese Hemodialysis Patients for Treatment of Anemia in Chronic Kidney Disease: SYMPHONY HD Study. Kidney Dis (Basel). 2021;7(6):494-502.

10.   Akizawa T, Nangaku M, Yamaguchi T, Koretomo R, Maeda K, Miyazawa Y, et al. A Phase 3 Study of Enarodustat in Anemic Patients with CKD not Requiring Dialysis: The SYMPHONY ND Study. Kidney Int Rep. 2021;6(7):1840-9.

11.	Akizawa T, Nangaku M, Yonekawa T, Okuda N, Kawamatsu S, Onoue T, et al. Efficacy and Safety of Daprodustat Compared with Darbepoetin Alfa in Japanese Hemodialysis Patients with Anemia: A Randomized, Double-Blind, Phase 3 Trial. Clin J Am Soc Nephrol. 2020;15(8):1155-65.

12.   Akizawa T, Yamada T, Nobori K, Matsuda Y, Hayashi Y, Hayasaki T, et al. Molidustat for Japanese Patients With Renal Anemia Receiving Dialysis. Kidney Int Rep. 2021;6(1):2604-16.

13.   Al-Ali FS, El-Sayed Abdelfattah M, Fawzy AA, Hamdy AF, Abdulla AE. Erythropoietin-stimulating agents in the management of anemia of end-stage renal disease patients on regular hemodialysis: a prospective randomized comparative study from Qatar. Hemodial Int. 2015;19(1):33-43.

14.   Al-Otaibi T, Gheith O, Halim MA, Abu Attia H, Mansour H, Said T, et al. Long-acting erythropoietin stimulating agents for persistent anemia after kidney transplant: risk factors and outcome. Exp Clin Transplant. 2014;12(3):220-6.

15.   Apsangikar P, Chaudhry S, Naik M, Deoghare S, Joseph J. Comparative efficacy and safety of biosimilar darbepoetin alfa in adults with anemia of chronic kidney disease. Indian Journal of Transplantation. 2018;12(1):30‐4.

16.   Argani H, Ossareh S, Tabiban S, Ganji MR, Nafar M, Abdi E, et al. PDpoietin in comparison with Eprex in treatment of anemic patients on hemodialysis. Iran J Kidney Dis. 2009;3(3):145‐50.

17.   Azmandian J, Abbasi MR, Pourfarziani V, Nasiri AA, Ossareh S, Ezzatzadegan Jahromi S, et al. Comparing Therapeutic Efficacy and Safety of Epoetin Beta and Epoetin Alfa in the Treatment of Anemia in End-Stage Renal Disease Hemodialysis Patients. Am J Nephrol. 2018;48(4):251-9.

18.   Baig MAA, Alam I, Sulehri FU, Hassan I, Jafri A, Butt HA. Change in Hemoglobin with Androgen and Low Dose Erythropoietin Versus Erythropoietin Alone in Patients of Anemia of Chronic Kidney Disease. Pakistan journal of medical and health sciences. 2021;15(1):3296‐8.

19.   Barratt J, Andric B, Tataradze A, Schömig M, Reusch M, Valluri U, et al. Roxadustat for the treatment of anaemia in chronic kidney disease patients not on dialysis: a Phase 3, randomized, open-label, active-controlled study (DOLOMITES). Nephrol Dial Transplant. 2021;36(9):1616-28.

20.   Beiraghdar F, Panahi Y, Einollahi B, Nemati E, Sahebkar A, Hassanzadeh A, et al. Evaluation of a biosimilar recombinant alpha epoetin in the management of anemia in hemodialysis patients. Saudi Pharm J. 2015;23(5):544-8.

21.   Beiraghdar F, Panahi Y, Einollahi B, Torkaman M, Mohammadi R, Marzony ET, et al. Investigation of the efficacy of a biogeneric recombinant human erythropoietin alfa in the correction of post-transplantation anemia: a randomized comparative trial with Eprex. Clin Lab. 2012;58(1):1179-85.

22.   Bernieh B, Abouchacra S, Boobes Y, Al Hakim MR, Nagelkerke N, Chaaban A, et al. Comparison between short- and long-acting erythropoiesis-stimulating agents in hemodialysis patients: target hemoglobin, variability, and outcome. Int Urol Nephrol. 2014;46(2):453-9.

23.   Brandt JR, Avner ED, Hickman RO, Watkins SL. Safety and efficacy of erythropoietin in children with chronic renal failure. Pediatr Nephrol. 1999;13(2):143-7.

24.   Campistol JM, Carreño A, Morales JM, Pallardó L, Franco A, Navarro D, et al. Once-monthly pegylated epoetin Beta versus darbepoetin alfa every two weeks in renal transplant recipients: a randomized trial. Transplantation. 2013;95(2):e6-e10.

25.   Carrera F, Lok CE, de Francisco A, Locatelli F, Mann JF, Canaud B, et al. Maintenance treatment of renal anaemia in haemodialysis patients with methoxy polyethylene glycol-epoetin beta versus darbepoetin alfa administered monthly: a randomized comparative trial. Nephrol Dial Transplant. 2010;25(1):4009-17.

26.   Charytan C, Manllo-Karim R, Martin ER, Steer D, Bernardo M, Dua SL, et al. A Randomized Trial of Roxadustat in Anemia of Kidney Failure: SIERRAS Study. Kidney Int Rep. 2021;6(7):1829-39.

27.   Chazot C, Terrat JC, Dumoulin A, Ang KS, Gassia JP, Chedid K, et al. Randomized equivalence study evaluating the possibility of switching hemodialysis patients receiving subcutaneous human erythropoietin directly to intravenous darbepoetin alfa. Annals of Pharmacotherapy. 2009;43(2):228-34.

28.   Chen N, Hao C, Liu BC, Lin H, Wang C, Xing C, et al. Roxadustat Treatment for Anemia in Patients Undergoing Long-Term Dialysis. N Engl J Med. 2019;381(1):1011-22.

29.   Chen N, Qian J, Chen J, Yu X, Mei C, Hao C, et al. Phase 2 studies of oral hypoxia-inducible factor prolyl hydroxylase inhibitor FG-4592 for treatment of anemia in China. Nephrol Dial Transplant. 2017;32(8):1373-86.

30.   Chen N, Qian JQ, Mei CL, Zhang AH, Xing CY, Wang L, et al. [The efficacy and safety of continuous erythropoietin receptor activator in dialytic patients with chronic renal anemia: an open, randomized, controlled, multi-center trial]. Zhonghua Nei Ke Za Zhi. 2012;51(7):502-7.

31.   Chen N, Xing C, Niu J, Liu B, Fu J, Zhao J, et al. Darbepoetin alfa injection versus epoetin alfa injection for treating anemia of Chinese hemodialysis patients with chronic kidney failure: A randomized, open-label, parallel-group, non-inferiority Phase III trail. Chronic Dis Transl Med. 2022;8(1):59-70.

32.   Chertow GM, Pergola PE, Farag YMK, Agarwal R, Arnold S, Bako G, et al. Vadadustat in Patients with Anemia and Non-Dialysis-Dependent CKD. N Engl J Med. 2021;384(1):1589-600.

33.   Choukroun G, Kamar N, Dussol B, Etienne I, Cassuto-Viguier E, Toupance O, et al. The complete correction of post-transplant anemia reduces the rate of progression of chronic allograft nephropathy. NDT Plus. 2010;3:iii267-iii8.

34.   Choukroun G, Renou M, Lecaque C, Jauréguy M. [TREAT or not to treat: anemia in type 2 diabetes and chronic kidney disease at stages 3 and 4]. Nephrol Ther. 2011;7(1):2-9.

35.   Coupes B, de Freitas DG, Roberts SA, Read I, Riad H, Brenchley PE, et al. rhErythropoietin-b as a tissue protective agent in kidney transplantation: a pilot randomized controlled trial. BMC Res Notes. 2015;8:21.

36.   Coyne DW. The health-related quality of life was not improved by targeting higher hemoglobin in the Normal Hematocrit Trial. Kidney Int. 2012;82(2):235-41.

37.   Coyne DW, Singh AK, Lopes RD, Bailey CK, DiMino TL, Huang C, et al. Three Times Weekly Dosing of Daprodustat versus Conventional Epoetin for Treatment of Anemia in Hemodialysis Patients: ASCEND-TD: A Phase 3 Randomized, Double-Blind, Noninferiority Trial. Clin J Am Soc Nephrol. 2022;17(9):1325-36.

38.   Csiky B, Schömig M, Esposito C, Barratt J, Reusch M, Valluri U, et al. Roxadustat for the Maintenance Treatment of Anemia in Patients with End-Stage Kidney Disease on Stable Dialysis: A European Phase 3, Randomized, Open-Label, Active-Controlled Study (PYRENEES). Adv Ther. 2021;38(1):5361-80.

39.   Do JH, Kim DJ, Choi SY, Do YS, Jang EH, Baek HJ. Efficacy and safety of high dose epoetin alfa therapy in CAPD patients by cross-over study. Korean journal of medicine. 2006;71(5):527‐34.

40.   Dwitanto K, Angginy N, Sutandar W. Comparative Study of Recombinant Human Erythropoietin (rhEPO) Products on CKD (Chronic Kidney Disease) Patients. Drug Res (Stuttg). 2023.

41.   Eckardt KU, Scherhag A, Macdougall IC, Tsakiris D, Clyne N, Locatelli F, et al. Left ventricular geometry predicts cardiovascular outcomes associated with anemia correction in CKD. J Am Soc Nephrol. 2009;20(12):2651-60.

42.   Eisenga MF, Emans ME, van der Putten K, Cramer MJ, Diepenbroek A, Velthuis BK, et al. Epoetin Beta and C-Terminal Fibroblast Growth Factor 23 in Patients With Chronic Heart Failure and Chronic Kidney Disease. J Am Heart Assoc. 2019;8(1):e011130.

43.   El-Ashmawy NE, Khedr EG, Kotb NS, Salem F, Ibrahim AO. Comparative Efficacy and Safety Study of Darbepoetin Alfa versus Epoetin Alfa in Management of Anemia Associated with ESRD in Egyptian Hemodialysis Patients. Curr Drug Saf. 2022;17(3):250-8.

44.   Fishbane S, Pollock CA, El-Shahawy M, Escudero ET, Rastogi A, Van BP, et al. Roxadustat Versus Epoetin Alfa for Treating Anemia in Patients with Chronic Kidney Disease on Dialysis: Results from the Randomized Phase 3 ROCKIES Study. J Am Soc Nephrol. 2022;33(4):850-66.

45.   Fishbane S, Singh B, Kumbhat S, Wisemandle WA, Martin NE. Intravenous Epoetin Alfa-epbx versus Epoetin Alfa for Treatment of Anemia in End-Stage Kidney Disease. Clin J Am Soc Nephrol. 2018;13(8):1204-14.

46.   Fishbane S, Spinowitz BS, Wisemandle WA, Martin NE. Randomized Controlled Trial of Subcutaneous Epoetin Alfa-epbx Versus Epoetin Alfa in End-Stage Kidney Disease. Kidney Int Rep. 2019;4(9):1235-47.

47.   Fliser D, Dellanna F, Koch M, Wiggenhauser A. Early low-dose erythropoiesis-stimulating agent therapy and progression of moderate chronic kidney disease: a randomized, placebo-controlled trial. Nephrol Dial Transplant. 2017;32(2):279-87.

48.   Foley RN, Curtis BM, Parfrey PS. Erythropoietin therapy, hemoglobin targets, and quality of life in healthy hemodialysis patients: A randomized trial. Clinical Journal of the American Society of Nephrology. 2009;4(4):726-33.

49.   Forni V, Bianchi G, Ogna A, Salvadé I, Vuistiner P, Burnier M, et al. Reticulocyte dynamic and hemoglobin variability in hemodialysis patients treated with Darbepoetin alfa and C.E.R.A.: a randomized controlled trial. BMC Nephrol. 2013;14:157.

50.   Furukawa T, Okada K, Abe M, Tei R, Oikawa O, Maruyama N, et al. Randomized Controlled Trial of Darbepoetin α Versus Continuous Erythropoietin Receptor Activator Injected Subcutaneously Once Every Four Weeks in Patients with Chronic Kidney Disease at the Pre-Dialysis Stage. Int J Mol Sci. 2015;16(1):30181-9.

51.   Gang S, Khetan P, Varade D, Chinta VR, Mavani S, Gupta U, et al. Desidustat in Anemia due to Dialysis-Dependent Chronic Kidney Disease: A Phase 3 Study (DREAM-D). Am J Nephrol. 2022;53(5):343-51.

52.   Gertz B, Kes P, Essaian A, Bias P, Buchner A, Zellner D. Epoetin theta: efficacy and safety of subcutaneous administration in anemic pre-dialysis patients in the maintenance phase in comparison to epoetin beta. Curr Med Res Opin. 2012;28(7):1101-10.

53.   Gertz B, Kohler E, Kes P, Essaian A, Bias P, Buchner A, et al. Epoetin theta: efficacy and safety of IV administration in anaemic haemodialysis patients in the maintenance phase in comparison to epoetin beta. Curr Med Res Opin. 2010;26(1):2393-402.

54.   Ghobrial EE, Salama KM, Shiba MF, El Shafae NH. The effect of anemia management on chronic renal failure progression. Egyptian pediatric association gazette. 2013;61(1):37‐41.

55.   Haag-Weber M, Eckardt KU, Hörl WH, Roger SD, Vetter A, Roth K. Safety, immunogenicity and efficacy of subcutaneous biosimilar epoetin-α (HX575) in non-dialysis patients with renal anemia: a multi-center, randomized, double-blind study. Clin Nephrol. 2012;77(1):8-17.

56.   Haag-Weber M, Vetter A, Thyroff-Friesinger U. Therapeutic equivalence, long-term efficacy and safety of HX575 in the treatment of anemia in chronic renal failure patients receiving hemodialysis. Clin Nephrol. 2009;72(5):380-90.

57.   Hafer C, Becker T, Kielstein JT, Bahlmann E, Schwarz A, Grinzoff N, et al. High-dose erythropoietin has no effect on short- or long-term graft function following deceased donor kidney transplantation. Kidney Int. 2012;81(3):314-20.

58.   Hayashi T, Maruyama S, Nangaku M, Narita I, Hirakata H, Tanabe K, et al. Darbepoetin Alfa in Patients with Advanced CKD without Diabetes: Randomized, Controlled Trial. Clin J Am Soc Nephrol. 2020;15(5):608-15.

59.   Hirakata H, Tsubakihara Y, Gejyo F, Nishi S, Iino Y, Watanabe Y, et al. Maintaining high hemoglobin levels improved the left ventricular mass index and quality of life scores in pre-dialysis Japanese chronic kidney disease patients. Clin Exp Nephrol. 2010;14(1):28-35.

60.   Holdstock L, Meadowcroft AM, Maier R, Johnson BM, Jones D, Rastogi A, et al. Four-Week Studies of Oral Hypoxia-Inducible Factor-Prolyl Hydroxylase Inhibitor GSK1278863 for Treatment of Anemia. J Am Soc Nephrol. 2016;27(4):1234-44.

61.   Hustrini NM, Siregar P, Setiawati A, Nugroho P. Clinical Comparison of Renogen, a Biosimilar Epoetin-a, with the Originator, Eprex, in Chronic Kidney Disease Anemia in Indonesia: A Preliminary Study. Acta Med Indones. 2019;51(3):230-7.

62.   Inrig JK, Barnhart HX, Reddan D, Patel UD, Sapp S, Califf RM, et al. Effect of hemoglobin target on progression of kidney disease: a secondary analysis of the CHOIR (Correction of Hemoglobin and Outcomes in Renal Insufficiency) trial. American Journal of Kidney Diseases. 2012;60(3):390‐401.

63.   Jang EH, Kang WH, Choi S. Time-averaged Concentration Hemoglobin and Erythropoietin Requirement According to Hemoglobin of Different Session of HD of the Week. Korean journal of nephrology. 2008;27(3):319‐26.

64.   Jehle PM, Jentzsch M, Langer T, Fröhling P, Fiedler R. Prospective 2-years study to evaluate malnutrition-inflammation- atherosclerosis (MIA) syndrome in hemodialysis patients: Inflammation-associated malnutrition is a significant predictor of mortality. Nieren- und Hochdruckkrankheiten. 2014;43(4):167-76.

65.   Johansen KL, Cobitz AR, Singh AK, Macdougall IC, Lopes RD, Obrador GT, et al. The ASCEND-NHQ randomized trial found positive effects of daprodustat on hemoglobin and quality of life in patients with non-dialysis chronic kidney disease. Kidney Int. 2023.

66.   Kakimoto-Shino M, Toya Y, Kuji T, Fujikawa T, Umemura S. Changes in hepcidin and reticulocyte hemoglobin equivalent levels in response to continuous erythropoietin receptor activator administration in hemodialysis patients: a randomized study. Ther Apher Dial. 2014;18(5):421-6.

67.   Kawai Y, Toya Y, Wakui H, Fujikawa T, Ueda E, Azushima K, et al. Potential effective treatment of shortening continuous erythropoietin receptor activator treatment interval combined with iron supplementation in hemodialysis patients. J Pharmacol Sci. 2021;147(1):118-25.

68.   Kawai Y, Toya Y, Wakui H, Fujikawa T, Ueda E, Azushima K, et al. Comparison of the effects of weekly and biweekly intravenous CERA administration on erythropoiesis: A randomized controlled trial. J Clin Hypertens (Greenwich). 2021;23(4):870-8.

69.   Keown PA, Churchill DN, Poulin-Costello M, Lei L, Gantotti S, Agodoa I, et al. Dialysis patients treated with Epoetin alfa show improved anemia symptoms: A new analysis of the Canadian Erythropoietin Study Group trial. Hemodial Int. 2010;14(2):168-73.

70.   Kessler M, Martínez-Castelao A, Siamopoulos KC, Villa G, Spinowitz B, Dougherty FC, et al. C.E.R.A. once every 4 weeks in patients with chronic kidney disease not on dialysis: The ARCTOS extension study. Hemodial Int. 2010;14(2):233-9.

71.   Kim CD, Park SH, Kim DJ, Park JW, Do JY, Shin SK, et al. Randomized trial to compare the dosage of darbepoetin alfa by administration route in haemodialysis patients. Nephrology. 2009;14(5):482-7.

72.   Kim DH, Lee YK, Kim J, Park HC, Yun KS, Cho A, et al. Effects of the route of erythropoietin administration on hemoglobin variability and cardiovascular events in hemodialysis patients. Kidney Research and Clinical Practice. 2021;40(4):724-33.

73.   Kim Y, Park SK, Cho WY, Joo KW, Shin SK, Kim DJ, et al. Efficacy and Safety of CKD-11101 (Proposed Biosimilar of Darbepoetin-Alfa) Compared with Darbepoetin-Alfa in Patients on Hemodialysis: A Randomized, Double-Blinded, Parallel-Group Phase III Study. BioDrugs. 2020;34(1):99-110.

74.   Kinugasa E, Yumita S, Sato T, Kurosawa A, Kitaoka T, Sugimoto H, et al. A dose-finding study of C. E. R. A. (Continuous Erythropoietin Receptor Activator) in renal anemia patients on hemodialysis - A randomized double-blind comparative study. Japanese pharmacology and therapeutics. 2011;39(SUPPL. 1):S9‐S19.

75.   Koiwa F, Suzuki E, Tomoyama H. Phase HI comparative study of darbepoetin alfa and biosimilar SK-1401 followed by subsequent long-term extension in renal anemia patients on hemodialysis. Japanese Pharmacology and Therapeutics. 2019;47(9):1393-406.

76.   Krivoshiev S, Wizemann V, Czekalski S, Schiller A, Pljesa S, Wolf-Pflugmann M, et al. Therapeutic equivalence of epoetin zeta and alfa, administered subcutaneously, for maintenance treatment of renal anemia. Adv Ther. 2010;27(2):105-17.

77.   Lee JY, Byun SW, Yeo YS, Yang WS, Park SK, Park JS. Comparison of Erythropoietic Effect between Epoetin-alpha and Darbepoetin-alpha in Hemodialysis Patients: a Randomized Crossover Study. Korean journal of nephrology. 2009;28(5):450‐5.

78.   Lee YK, Koo JR, Kim JK, Park II, Joo MH, Yoon JW, et al. Effect of Route of EPO Administration on Hemodialysis Arteriovenous Vascular Access Failure: A Randomized Controlled Trial. American Journal of Kidney Diseases. 2009;53(5):815-22.

79.   Lee YK, Lee HS, Joo MH, Kim SG, Seo JW, Oh JE. The Efficacy of Once Weekly Compared with Two or Three Times Weekly Subcutaneous Epoetin Alfa (Espogen(R)). Korean journal of nephrology. 2007;26(2):220‐6.

80.   Levin NW, Fishbane S, Cañedo FV, Zeig S, Nassar GM, Moran JE, et al. Intravenous methoxy polyethylene glycol-epoetin beta for haemoglobin control in patients with chronic kidney disease who are on dialysis: a randomised non-inferiority trial (MAXIMA). Lancet (london, england). 2007;370(9):1415‐21.

81.   Lewis EF, Pfeffer MA, Feng A, Uno H, McMurray JJ, Toto R, et al. Darbepoetin alfa impact on health status in diabetes patients with kidney disease: a randomized trial. Clinical Journal of the American Society of Nephrology. 2011;6(4):845‐55.

82.   Liao SC, Hung CC, Lee CT, Lee CH, Lee CC, Lin CL, et al. Switch From Epoetin Beta to Darbepoetin Alfa Treatment of Anemia in Taiwanese Hemodialysis Patients: Dose Equivalence by Hemoglobin Stratification. Therapeutic Apheresis and Dialysis. 2016;20(4):400-7.

83.   Lim SK, Goh BL, Visvanathan R, Kim SH, Jeon JS, Kim SG, et al. A multicentre, multi-national, double-blind, randomised, active-controlled, parallel-group clinical study to assess the safety and efficacy of PDA10 (Epoetin-alpha) vs. Eprex® in patients with anaemia of chronic renal failure. BMC Nephrol. 2021;22(1):391.

84.   Liu B, Chen N, Zhao J, Yin A, Wu X, Xing C, et al. Efficacy and safety of darbepoetin alfa injection replacing epoetin alfa injection for the treatment of renal anemia in Chinese hemodialysis patients: A randomized, open-label, parallel-group, noninferiority phase III trial. Chronic Dis Transl Med. 2022;8(2):134-44.

85.   Locatelli F, Hannedouche T, Fishbane S, Morgan Z, Oguey D, White WB. Cardiovascular Safety and All-Cause Mortality of Methoxy Polyethylene Glycol-Epoetin Beta and Other Erythropoiesis-Stimulating Agents in Anemia of CKD: A Randomized Noninferiority Trial. Clin J Am Soc Nephrol. 2019;14(1):1701-10.

86.   Locatelli F, Mann JF, Aldigier JC, Sanz Guajardo D, Schmidt R, Van Vlem B, et al. C.E.R.A. safety profile: a pooled analysis in patients with chronic kidney disease. Clin Nephrol. 2010;73(2):94-103.

87.   Lui SF, Chung WW, Leung CB, Chan K, Lai KN. Pharmacokinetics and pharmacodynamics of subcutaneous and intraperitoneal administration of recombinant human erythropoietin in patients on continuous ambulatory peritoneal dialysis. Clin Nephrol. 1990;33(1):47‐51.

88.   Macdougall IC, Akizawa T, Berns JS, Bernhardt T, Krueger T. Effects of Molidustat in the Treatment of Anemia in CKD. Clin J Am Soc Nephrol. 2019;14(1):28-39.

89.   Macdougall IC, Provenzano R, Sharma A, Spinowitz BS, Schmidt RJ, Pergola PE, et al. Peginesatide for anemia in patients with chronic kidney disease not receiving dialysis. N Engl J Med. 2013;368(4):320-32.

90.   Macdougall IC, Walker R, Provenzano R, de Alvaro F, Locay HR, Nader PC, et al. C.E.R.A. corrects anemia in patients with chronic kidney disease not on dialysis: results of a randomized clinical trial. Clin J Am Soc Nephrol. 2008;3(2):337-47.

91.   Mann JFE, de Francisco A, Nassar G, Canaud B. Fewer dose changes with once-monthly C.E.R.A. in patients with chronic kidney disease. Clin Nephrol. 2011;76(1):9‐15.

92.   Martin KJ. The first human cell line-derived erythropoietin, epoetin-δ (Dynepo®), in the management of anemia in patients with chronic kidney disease. Nieren- und Hochdruckkrankheiten. 2008;37(1):38‐45.

93.   Maruyama S, Kurasawa S, Hayashi T, Nangaku M, Narita I, Hirakata H, et al. Higher hemoglobin levels using darbepoetin alfa and kidney outcomes in advanced chronic kidney disease without diabetes: a prespecified secondary analysis of the PREDICT trial. Clin Exp Nephrol. 2023;27(9):757-66.

94.   Mazahir R, Anand K, Pruthi PK. Comparison of darbepoetin alpha and recombinant human erythropoietin for treatment of anemia in pediatric chronic kidney disease: a non-inferiority trial from India. Eur J Pediatr. 2023;182(1):101-9.

95.   Mc Causland FR, Claggett B, Burdmann EA, Chertow GM, Cooper ME, Eckardt KU, et al. Treatment of Anemia With Darbepoetin Prior to Dialysis Initiation and Clinical Outcomes: Analyses From the Trial to Reduce Cardiovascular Events With Aranesp Therapy (TREAT). Am J Kidney Dis. 2019;73(3):309-15.

96.   McMurray JJ, Uno H, Jarolim P, Desai AS, de Zeeuw D, Eckardt KU, et al. Predictors of fatal and nonfatal cardiovascular events in patients with type 2 diabetes mellitus, chronic kidney disease, and anemia: an analysis of the Trial to Reduce cardiovascular Events with Aranesp (darbepoetin-alfa) Therapy (TREAT). Am Heart J. 2011;162(4):748-55.e3.

97.   Miao B, Isachkina AN, Shutov EV, Selyutin AA, Kvitkova LV, Shilo VY, et al. Biosimilar erythropoietin in anemia treatment (BEAT)-Efficacy and safety of a 1:1 dose conversion from EPREX® to EPIAO® in patients with end-stage renal disease on hemodialysis: A prospective, randomized, double blind, parallel group study. Medicine (Baltimore). 2022;101(4):e31426.

98.   Morris KP, Skinner JR, Hunter S, Coulthard MG. Short term correction of anaemia with recombinant human erythropoietin and reduction of cardiac output in end stage renal failure. Archives of disease in childhood. 1993;68(5):644-8.

99.   Muirhead N, Keown PA, Churchill DN, Poulin-Costello M, Gantotti S, Lei L, et al. Dialysis patients treated with Epoetin α show improved exercise tolerance and physical function: A new analysis of the Canadian Erythropoietin Study Group trial. Hemodial Int. 2011;15(1):87-94.

100. Na HY, Lee YK, Shin SK, Yang DH, Cheon W, Park JH, et al. A randomized crossover study of single biweekly administration of epoetin-alpha compared with darbepoetin-alpha in chronic kidney disease patients not receiving dialysis. Kidney Research and Clinical Practice. 2014;33(4):210‐6.

101. Nagaya H, Inaguma D, Kitagawa A, Murata M, Kamimura Y, Hamaguchi K, et al. Intravenously administered darbepoetin alfa once a week could maintain hemoglobin level more efficiently than once every 2 weeks in patients on hemodialysis. Clin Exp Nephrol. 2010;14(2):158-63.

102. Nand N, Nandan R, Jain D. Comparative Effects of Pegylated Erythropoietin and Darbepoetin Alfa on Erythropoietin Hyporesponsive Anemia of Patients with Chronic Kidney Disease on Maintenance Hemodialysis. J Assoc Physicians India. 2021;69(1):11-2.

103. Nangaku M, Hamano T, Akizawa T, Tsubakihara Y, Nagai R, Okuda N, et al. Daprodustat Compared with Epoetin Beta Pegol for Anemia in Japanese Patients Not on Dialysis: A 52-Week Randomized Open-Label Phase 3 Trial. Am J Nephrol. 2021;52(1):26-35.

104. Nangaku M, Kondo K, Kokado Y, Ueta K, Kaneko G, Tandai T, et al. Phase 3 Randomized Study Comparing Vadadustat with Darbepoetin Alfa for Anemia in Japanese Patients with Nondialysis-Dependent CKD. J Am Soc Nephrol. 2021;32(7):1779‐90.

105. Nangaku M, Kondo K, Ueta K, Kokado Y, Kaneko G, Matsuda H, et al. Efficacy and safety of vadadustat compared with darbepoetin alfa in Japanese anemic patients on hemodialysis: a Phase 3, multicenter, randomized, double-blind study. Nephrol Dial Transplant. 2021;36(9):1731-41.

106. Nishi S, Yamada M, Tsuruya K, Masakane I, Nakamoto H. JR-131, a Biosimilar of Darbepoetin Alfa, for the Treatment of Hemodialysis Patients With Renal Anemia: A Randomized, Double-Blinded, Parallel-Group Phase 3 Study. Ther Apher Dial. 2020;24(2):126-35.

107. Oh J, Joo KW, Chin HJ, Chae DW, Kim SG, Kim SJ, et al. Correction of anemia with continuous erythropoietin receptor activator in Korean patients on long-term hemodialysis. J Korean Med Sci. 2014;29(1):76-83.

108. Ohki K, Wakui H, Uneda K, Azushima K, Haruhara K, Kinguchi S, et al. Effects of Erythropoietin-Stimulating Agents on Blood Pressure in Patients with Non-Dialysis CKD and Renal Anemia. Kidney Dis (Basel). 2020;6(4):299-308.

109. Ostrvica E, Mesic E, Ostrvica D, Delic J, Delic-Custendil S, Hukic F. Effectiveness of treating the renal anemia in chronic hemodialyzed patients by epoietin alpha and beta. Med Arh. 2010;64(1):4-6.

110.  Otsuka T, Sakai Y, Yui S, Sukegawa M, Suzuki A, Mugishima K, et al. Comparison of pain and efficacy of darbepoetin alfa and epoetin Beta pegol treatment in patients receiving peritoneal dialysis. J Nippon Med Sch. 2015;82(1):21-6.

111.  Palazzuoli A, Quatrini I, Calabrò A, Antonelli G, Caputo M, Campagna MS, et al. Anemia correction by erythropoietin reduces BNP levels, hospitalization rate, and NYHA class in patients with cardio-renal anemia syndrome. Clinical and experimental medicine. 2011;11(1):43-8.

112.  Patel M, Thimons DG, Winston JL, Langholff W, McGowan T. An open-label, randomized, multicenter, controlled study of epoetin alfa for the treatment of anemia of chronic kidney disease in the long term care setting. J Am Med Dir Assoc. 2012;13(3):244-8.

113.  Patel T, Hirter A, Kaufman J, Keithi-Reddy SR, Reda D, Singh A. Route of epoetin administration influences hemoglobin variability in hemodialysis patients. Am J Nephrol. 2009;29(6):532-7.

114.  Pergola PE, Gartenberg G, Fu M, Sun S, Wolfson M, Bowers P. A randomized controlled study comparing once-weekly to every-2-week and every-4-week dosing of epoetin alfa in CKD patients with anemia. Clinical Journal of the American Society of Nephrology. 2010;5(4):598‐606.

115.  Pergola PE, Gartenberg G, Fu M, Wolfson M, Rao S, Bowers P. A randomized controlled study of weekly and biweekly dosing of epoetin alfa in CKD Patients with anemia. Clin J Am Soc Nephrol. 2009;4(1):1731-40.

116.  Pfeffer MA, Burdmann EA, Chen CY, Cooper ME, de Zeeuw D, Eckardt KU, et al. A trial of darbepoetin alfa in type 2 diabetes and chronic kidney disease. N Engl J Med. 2009;361(2):2019-32.

117.  Picon PD, Pribbernow SC, Prompt CA, Schacher SC, Antunes VV, Mentz BP, et al. Randomized double-blind clinical trial of a new human epoetin versus a commercially available formula for anemia control in patients on hemodialysis. Clinics (Sao Paulo). 2014;69(8):547-53.

118.  Pile T, Raftery M, Thuraisingham R, Kirwan CJ, Harwood S, Yaqoob MM. Treating Posttransplant Anemia With Erythropoietin Improves Quality of Life but Does Not Affect Progression of Chronic Kidney Disease. Exp Clin Transplant. 2020;18(1):27-33.

119.  Provenzano R, Besarab A, Macdougall IC, Ellison DH, Maxwell AP, Sulowicz W, et al. The continuous erythropoietin receptor activator (C.E.R.A.) corrects anemia at extended administration intervals in patients with chronic kidney disease not on dialysis: results of a phase II study. Clin Nephrol. 2007;67(5):306‐17.

120. Provenzano R, Besarab A, Wright S, Dua S, Zeig S, Nguyen P, et al. Roxadustat (FG-4592) Versus Epoetin Alfa for Anemia in Patients Receiving Maintenance Hemodialysis: A Phase 2, Randomized, 6- to 19-Week, Open-Label, Active-Comparator, Dose-Ranging, Safety and Exploratory Efficacy Study. Am J Kidney Dis. 2016;67(6):912-24.

121. Provenzano R, Shutov E, Eremeeva L, Korneyeva S, Poole L, Saha G, et al. Roxadustat for anemia in patients with end-stage renal disease incident to dialysis. Nephrol Dial Transplant. 2021;36(9):1717-30.

122. Provenzano R, Singh AK. Hemoglobin maintenance with use of extended dosing of epoetin alfa in patients with diabetes and anemia of chronic kidney disease. Endocrine Practice. 2007;13(3):251‐9.

123. Roger SD, Jassal SV, Woodward MC, Soroka S, McMahon LP. A randomised single-blind study to improve health-related quality of life by treating anaemia of chronic kidney disease with Aranesp® (darbepoetin alfa) in older people: STIMULATE. Int Urol Nephrol. 2014;46(2):469‐75.

124. Roger SD, Kolmakova E, Fung M, Malecki R, Vinhas J, Dellanna F, et al. Darbepoetin alfa once monthly corrects anaemia in patients with chronic kidney disease not on dialysis. Nephrology (Carlton). 2014;19(5):266-74.

125. Roger SD, Locatelli F, Woitas RP, Laville M, Tobe SW, Provenzano R, et al. C.E.R.A. once every 4 weeks corrects anaemia and maintains haemoglobin in patients with chronic kidney disease not on dialysis. Nephrol Dial Transplant. 2011;26(1):3980-6.

126. Saglimbene V, Palmer SC, Craig JC, Ruospo M, Nicolucci A, Tonelli M, et al. Low versus high dose erythropoiesis-stimulating agents in hemodialysis patients with anemia: A randomized clinical trial. PLoS One. 2017;12(3):e0172735.

127. Satirapoj B, Dispan R, Supasyndh O. Efficacy and safety of subcutaneous administration of lyophilized powder of alfa-erythropoietin to maintain hemoglobin concentrations among hemodialysis patients. International Journal of Nephrology and Renovascular Disease. 2017;10:275-83.

128. Satirapoj B, Supasyndh O, Choovichian P. A comparative study of efficacy and safety of the lyophilized powder alpha-erythropoietin and the liquid form alpha-erythropoietin for hemoglobin maintenance in patients with hemodialysis treatment. J Med Assoc Thai. 2014;97(9):899-906.

129. Singh AK, Carroll K, McMurray JJV, Solomon S, Jha V, Johansen KL, et al. Daprodustat for the Treatment of Anemia in Patients Not Undergoing Dialysis. N Engl J Med. 2021;385(2):2313-24.

130. Singh AK, Carroll K, Perkovic V, Solomon S, Jha V, Johansen KL, et al. Daprodustat for the Treatment of Anemia in Patients Undergoing Dialysis. N Engl J Med. 2021;385(2):2325-35.

131. Singh AK, Cizman B, Carroll K, McMurray JJV, Perkovic V, Jha V, et al. Efficacy and Safety of Daprodustat for Treatment of Anemia of Chronic Kidney Disease in Incident Dialysis Patients: A Randomized Clinical Trial. JAMA Intern Med. 2022;182(6):592-602.

132. Sinha SD, Bandi VK, Bheemareddy BR, Thakur P, Chary S, Mehta K, et al. Efficacy, tolerability and safety of darbepoetin alfa injection for the treatment of anemia associated with chronic kidney disease (CKD) undergoing dialysis: a randomized, phase-III trial. BMC Nephrol. 2019;20(1):90.

133. Skali H, Parving HH, Parfrey PS, Burdmann EA, Lewis EF, Ivanovich P, et al. Stroke in patients with type 2 diabetes mellitus, chronic kidney disease, and anemia treated with Darbepoetin Alfa: the trial to reduce cardiovascular events with Aranesp therapy (TREAT) experience. Circulation. 2011;124(2):2903‐8.

134. Song HY, Yoon HS, Kang SW, Choi KH, Ha SK, Lee HY, et al. Comparative prospective study of intravenously and subcutaneously administered recombinant human erythropoietin(epokine(r)) in end-stage renal disease patients : a phase IV single center study. Korean journal of nephrology. 2002;21(2):190‐8.

135. Spinowitz B, Coyne DW, Lok CE, Fraticelli M, Azer M, Dalal S, et al. C.E.R.A. maintains stable control of hemoglobin in patients with chronic kidney disease on dialysis when administered once every two weeks. Am J Nephrol. 2008;28(2):280‐9.

136. Steffensen GK, Stergaard O. Administration of the same dose of epoetin-beta intravenously and subcutaneously to patients with renal anaemia. Scand J Urol Nephrol. 2011;45(6):461-9.

137. Suzuki M, Hada Y, Akaishi M, Hiroe M, Aonuma K, Tsubakihara Y, et al. Effects of anemia correction by erythropoiesis-stimulating agents on cardiovascular function in non-dialysis patients with chronic kidney disease. Int Heart J. 2012;53(4):238-43.

138. Teruel JL, Sanchez FJL, Ortuno J. Pain after subcutaneous inyection of erythropoietin: comparison between the new formulation of alpha erythropoietin and beta erythropoietin. Nefrologia. 1997;17(3):214‐20.

139. Thadhani R, Guilatco R, Hymes J, Maddux FW, Ahuja A. Switching from Epoetin Alfa (Epogen) to Epoetin Alfa-Epbx (RetacritTM) Using a Specified Dosing Algorithm: a Randomized, Non-Inferiority Study in Adults on Hemodialysis. Am J Nephrol. 2018:214‐24.

140. Toida T, Sato Y, Shibata N, Kitamura K, Fujimoto S. A randomized control study on the procedure for switching epoetin beta (EPO) to epoetin beta pegol (CERA) in the treatment of renal anemia in maintenance hemodialysis patients. Blood Purif. 2014;38(3):174-9.

141. Toto R, Petersen J, Berns JS, Lewis EF, Tran Q, Weir MR. A Randomized Trial of Strategies Using Darbepoetin Alfa To Avoid Transfusions in CKD. J Am Soc Nephrol. 2021;32(2):469‐78.

142. Tsubakihara Y, Akizawa T, Iwasaki M, Shimazaki R. High Hemoglobin Levels Maintained by an Erythropoiesis-Stimulating Agent Improve Renal Survival in Patients with Severe Renal Impairment. Ther Apher Dial. 2015;19(5):457-65.

143. Tsubakihara Y, Gejyo F, Nishi S, Iino Y, Watanabe Y, Suzuki M, et al. High target hemoglobin with erythropoiesis-stimulating agents has advantages in the renal function of non-dialysis chronic kidney disease patients. Ther Apher Dial. 2012;16(6):529-40.

144. Tsuruya K, Hayashi T, Yamamoto H, Hase H, Nishi S, Yamagata K, et al. Renal prognoses by different target hemoglobin levels achieved by epoetin beta pegol dosing to chronic kidney disease patients with hyporesponsive anemia to erythropoiesis-stimulating agent: a multicenter open-label randomized controlled study. Clin Exp Nephrol. 2021;25(5):456-66.

145. Uriol-Rivera MG, Obrador-Mulet A, Jimenez-Mendoza S, Corral-Baez A, Perianez-Parraga L, Garcia-Alvarez A, et al. Monthly Continuous Erythropoietin Receptor Activator Versus Weekly Epoetin-Beta, Similar Hemoglobinization but Different Anisocytosis Degree in Hemodialysis Patients: A Randomized Controlled Trial. J Hematol. 2021;10(6):255-65.

146. van der Putten K, Jie KE, van den Broek D, Kraaijenhagen RJ, Laarakkers C, Swinkels DW, et al. Hepcidin-25 is a marker of the response rather than resistance to exogenous erythropoietin in chronic kidney disease/chronic heart failure patients. European journal of heart failure. 2010;12(9):943‐50.

147. Vuksanović-Mikulicić S, Mikolasević I, Jelić I, Bubić I, Sladoje-Martinović B, Racki S. [Clinical relevance of anemia treatment in patients with chronic kidney disease]. Acta Med Croatica. 2012;66(3):193-202.

148. Warady BA, Arar MY, Lerner G, Nakanishi AM, Stehman-Breen C. Darbepoetin alfa for the treatment of anemia in pediatric patients with chronic kidney disease. Pediatr Nephrol. 2006;21(8):1144-52.

149. Warady BA, Barcia J, Benador N, Jankauskiene A, Olson K, Podracka L, et al. De novo weekly and biweekly darbepoetin alfa dosing in pediatric patients with chronic kidney disease. Pediatr Nephrol. 2018;33(1):125-37.

150. Weir MR, Pergola PE, Agarwal R, Fink JC, Kopyt NP, Singh AK, et al. A Comparison of the Safety and Efficacy of HX575 (Epoetin Alfa Proposed Biosimilar) with Epoetin Alfa in Patients with End-Stage Renal Disease. Am J Nephrol. 2017;46(5):364-70.

151. Woodland AL, Murphy SW, Curtis BM, Barrett BJ. Costs Associated With Intravenous Darbepoetin Versus Epoetin Therapy in Hemodialysis Patients: A Randomized Controlled Trial. Can J Kidney Health Dis. 2017;4:2054358117716461.

152. Yalçinkaya F, Tümer N, Cakar N, Ozkaya N. Low-dose erythropoietin is effective and safe in children on continuous ambulatory peritoneal dialysis. Pediatr Nephrol. 1997;11(3):350-2.

153. Yamamoto H, Nobori K, Matsuda Y, Hayashi Y, Hayasaki T, Akizawa T. Efficacy and Safety of Molidustat for Anemia in ESA-Naive Nondialysis Patients: A Randomized, Phase 3 Trial. Am J Nephrol. 2021;52(1):871-83.

154. Yamamoto H, Nobori K, Matsuda Y, Hayashi Y, Hayasaki T, Akizawa T. Molidustat for Renal Anemia in Nondialysis Patients Previously Treated with Erythropoiesis-Stimulating Agents: A Randomized, Open-Label, Phase 3 Study. Am J Nephrol. 2021;52(1):884-93.

155. Yamazaki C, Watanabe Y, Sakamoto N. Pharmacokinetic study of recombinant human erythropoietin treatment in pre-dialysis end stage renal disease patients. Nihon Jinzo Gakkai shi. 1993;35(1):1233‐42.

156. Yasari F, Nafar M, Alipour Abdei B, Ahmadpoor P, Pour-Reza-Gholi F, Samadian F, et al. Effect of erythropoietin on kidney allograft survival: early use after transplantation. Iran J Kidney Dis. 2012;6(1):44-8.

157. Yeo HM, Kim DJ, Choi SY, Do YS, Jang EH, Baek HJ. Effect of High Dose (10, 000 IU) Epoetin Alfa (Espogen(R)) Therapy in CAPD Patients. Korean journal of nephrology. 2005;24(3):441‐7.

158. Yoon SY, Choi BS, Yang CW, Kim YS, Bang BK, Joo KW. Randomized, Controlled Trial of Darbepoetin Alfa for the Treatment of Renal Anemia in Hemodialysis Patients. Korean journal of nephrology. 2005;24(3):429‐40.

159. Zhang P, Jiang Y, Xu C, Zhou L, Zheng H, Xie D, et al. Pegmolesatide for the treatment of anemia in patients undergoing dialysis: a randomized clinical trial. EClinicalMedicine. 2023;65:102273.
